# Growth Hormone (GH) Enhances Endogenous Mechanisms of Neuroprotection and Neuroplasticity after Oxygen and Glucose Deprivation Injury (OGD) and Reoxygenation (OGD/R) in Chicken Hippocampal Cell Cultures

**DOI:** 10.1155/2021/9990166

**Published:** 2021-09-16

**Authors:** Juan David Olivares-Hernández, Jerusa Elienai Balderas-Márquez, Martha Carranza, Maricela Luna, Carlos G. Martínez-Moreno, Carlos Arámburo

**Affiliations:** Departamento de Neurobiología Celular y Molecular, Instituto de Neurobiología, Universidad Nacional Autónoma de México, Campus Juriquilla, Querétaro, Qro., 76230, Mexico

## Abstract

As a classical growth promoter and metabolic regulator, growth hormone (GH) is involved in development of the central nervous system (CNS). This hormone might also act as a neurotrophin, since GH is able to induce neuroprotection, neurite growth, and synaptogenesis during the repair process that occurs in response to neural injury. After an ischemic insult, the neural tissue activates endogenous neuroprotective mechanisms regulated by local neurotrophins that promote tissue recovery. In this work, we investigated the neuroprotective effects of GH in cultured hippocampal neurons exposed to hypoxia-ischemia injury and further reoxygenation. Hippocampal cell cultures obtained from chick embryos were incubated under oxygen-glucose deprivation (OGD, <5% O_2_, 1 g/L glucose) conditions for 24 h and simultaneously treated with GH. Then, cells were either collected for analysis or submitted to reoxygenation and normal glucose incubation conditions (OGD/R) for another 24 h, in the presence of GH. Results showed that OGD injury significantly reduced cell survival, the number of cells, dendritic length, and number of neurites, whereas OGD/R stage restored most of those adverse effects. Also, OGD/R increased the mRNA expression of several synaptogenic markers (i.e., NRXN1, NRXN3, NLG1, and GAP43), as well as the growth hormone receptor (GHR). The expression of BDNF, IGF-1, and BMP4 mRNAs was augmented in response to OGD injury, and exposure to OGD/R returned it to normoxic control levels, while the expression of NT-3 increased in both conditions. The addition of GH (10 nM) to hippocampal cultures during OGD reduced apoptosis and induced a significant increase in cell survival, number of cells, and doublecortin immunoreactivity (DCX-IR), above that observed in the OGD/R stage. GH treatment also protected dendrites and neurites during OGD, inducing plastic changes reflected in an increase and complexity of their outgrowths during OGD/R. Furthermore, GH increased the expression of NRXN1, NRXN3, NLG1, and GAP43 after OGD injury. GH also increased the BDNF expression after OGD, but reduced it after OGD/R. Conversely, BMP4 was upregulated by GH after OGD/R. Overall, these results indicate that GH protective actions in the neural tissue may be explained by a synergic combination between its own effect and that of other local neurotrophins regulated by autocrine/paracrine mechanisms, which together accelerate the recovery of tissue damaged by hypoxia-ischemia.

## 1. Introduction

Ischemic stroke is a serious cerebrovascular event caused by a blockage of blood supply and oxygen to the brain, leading to damage or death of brain cells, which produces a severe neurological impairment, or even decease [1]. It is well established that cerebral ischemia induces a pathophysiological response in the neural tissue that leads to apoptotic and necrotic cell death [2], neural structural damage, and synaptic loss, which then contribute to the drastic deficiency of neurological functions [3].

In addition to its classical actions on growth and metabolism, growth hormone (GH) has been reported to play a relevant role, as a neurotrophic factor, on brain repair after traumatic brain injury (TBI) and stroke [4–6]. The neurotrophic actions of GH in the central nervous system (CNS) include prosurvival effects during embryonic development [7, 8], neurogenesis in the adult brain [9], structural plasticity [10, 11], and synaptogenesis [12], among others. These effects could be associated with the cognitive and motor improvement observed in TBI patients, with or without growth hormone deficiency (GHD), who received GH therapy [6, 13–15].

It has been reported that after neural injury, there is an activation of local mechanisms that induce neuroprotection and neuroplasticity which, in some cases, also promote proliferation of newly born neurons and migration of neural precursor cells into the lesioned peri-infarct region [16, 17]. The cellular and molecular mechanisms behind the brain capacity to repair an infarcted region are still largely undetermined; although, the expression and release of endogenous neurotrophic factors have been shown to be significantly increased during ischemic events [18–21]. Interestingly, GH is also synthesized by cells surrounding the peri-infarcted area suggesting that local autocrine/paracrine mechanisms are triggered after a neural injury [22]. Moreover, it has been shown that the expression of growth hormone receptor (GHR) is increased in the injured tissue, facilitating the neuroprotective action of this hormone [23].

Neuroprotective actions of GH treatments on either brain ischemia in vivo or oxygen-glucose deprivation (OGD) injury in vitro have been previously documented [24–26]. In the hippocampus, GH significantly reduced apoptotic cell death rate after an experimental stroke [25], decreased loss of neural tissue, and increased the expression of neurotrophic factors, synaptogenesis, and myelination biomarkers, as well as the formation of new blood vessels within the peri-infarct area, and provoked an improvement in cognitive function in experimentally stroked mice [26]. Recent studies showed that the administration of GH after experimental stroke promoted neurogenesis and stimulated synaptic plasticity and angiogenesis within the peri-infarct region, which were associated with an improvement in the motor function [27]. Furthermore, GH treatment promoted remote hippocampal plasticity and enhanced cognitive recovery after cortical injury [28]. Relevantly, GH addition also increased the expression of neurotrophic factors, such as BDNF and IGF-1, which in turn could participate in the endogenous neuroprotective response that occurs after ischemic injury [4, 25, 29].

Given the increasing number of reports regarding the beneficial effects of GH treatment in patients with brain injury and stroke [4, 5, 14, 30–32], as well as its therapeutic potential to treat neurodegenerative diseases [33, 34], it is pertinent to further investigate the interactions between the administration of GH and the expression of endogenous neurotrophic factors that may be involved in local neuroprotection mechanisms. Thus, the aim of the present study was to evaluate the neuroprotective role of GH in cultured hippocampal neurons that were injured by exposition to OGD and then submitted to an additional reoxygenation (OGD/R) period.

This work shows that OGD injury (24 h) significantly affects cell survival, reduces neurite outgrowth, and alters the expression of several synaptogenic markers, such as neurexins (NRXN1, NRXN3), neuroligins (NLG1), and growth associated protein 43 (GAP43), in hippocampal neurons. Interestingly, exposure of the harmed cultures to reoxygenation and normal glucose incubation conditions (OGD/R), for another 24 h, reverses most of the adverse effects of OGD. Additionally, it is shown that the expression of several neurotrophic factors (i.e., BDNF, NT-3, IGF-1, and BMP4) is significantly increased after OGD, whereas the GHR expression was upregulated only in the OGD/R condition. Furthermore, it was found that administration of GH treatments, both under OGD and OGD/R conditions, clearly stimulated significant plastic changes by promoting cell survival, enabling an increase of neurite outgrowth, and inducing a rise of synapse formation markers, in levels above those observed in the OGD/R condition. These protective neurotrophic actions of GH are probably mediated through a synergistic mechanism between GH and other endogenous neurotrophins.

## 2. Materials and Methods

### 2.1. Animals

Fertilized eggs (*Gallus gallus*, White Leghorn) were obtained from Pilgrim's Pride (Querétaro, México) and were incubated at 39°C in a humidified air chamber (IAMEX, México) until embryonic day 15 (ED15). The eggs were rotated one quarter of a revolution every 50 min during incubation. All experimental procedures were conducted according to national (NOM-062-ZOO-1999) and international (*The guide for care and use of laboratory animals*, U.S. National Research Council) guidelines and were approved by the bioethical committee of the Instituto de Neurobiología, UNAM.

### 2.2. Embryonic Chicken Hippocampal Cell Cultures

Chick embryos (ED15) were anesthetized in ovo by cooling them on ice for 5 min and then euthanized by decapitation. The brain was pulled out quickly from the skull, and it was coronally chopped into a block, at the level of hippocampus using a microsurgical blade. The slices were examined under the stereoscopic microscope, and the hippocampi were microdissected, using the lateral ventricles as reference. The hippocampal tissues from 15 embryos were digested in 0.002% trypsin-EDTA (Sigma-Aldrich, St. Louis, MO, USA) solution at 39°C for 10 min. Trypsin digestion was stopped by the addition of an equal volume of neurobasal medium supplemented with 2% B27, GlutaMAX, and 1% penicillin-streptomycin (Gibco, Grand Island, NY, USA). The digested tissue was centrifuged at 1800 g for 5 min, and the pellet was resuspended in the same medium. The tissue was mechanically triturated with a glass pipette and passed through a 40 *μ*m pore filter. Cells were counted in a Neubauer chamber using the trypan blue exclusion method, and then (1 × 10^6^) plated in 12-well plates (Costar Corning, NY, USA) coated with 50 *μ*g/mL poly-L-lysine, and incubated in neurobasal-B27 (Nb-B27) media. The cell cultures were stabilized in a humidified incubator at 39°C, under an atmosphere of 95% air/5% CO_2_, for 5 days.

### 2.3. Treatments

An in vitro model of cerebral ischemia/reperfusion was established by incubating hippocampal cell cultures first under oxygen-glucose deprivation (OGD) and then under reoxygenation (OGD/R). OGD was induced by replacing Nb-B27 medium for DMEM-Low glucose 1× (I g/L, Gibco, Grand Island, NY, USA) medium; then, the hippocampal cell cultures were incubated in a humidified hypoxic chamber (Napco E Series, Model 302 CO_2_ incubator), for 24 h at 37°C, and flushed with a 95% N_2_/5% CO_2_ gas mixture. The resulting oxygen concentration within the chamber (<5% O_2_) was monitored with an ambient oxygen sensor (BW Technologies, Arlington, TX, USA) and maintained throughout the experiment. Immediately after this treatment, a group of cells were used as hypoxia-ischemia controls (ODG group) and analyzed, whereas others were exposed to reoxygenation for another 24 h (OGD/R group). The OGD/R condition was achieved by substituting DMEM-Low glucose medium for Nb-B27 medium and incubating the cultures for additional 24 h under normoxic conditions (95% air/5% CO_2_). After the OGD/R stage, the cell cultures were used for analysis. As the control group, cell cultures were maintained in normal Nb-B27 medium under normoxic conditions (normoxia group).

Recombinant chicken growth hormone (rcGH, Revholt, PRL, Israel) was administered to the cell cultures in DMEM-Low glucose medium or Nb-B27 medium at several concentrations (1, 10, 100 nM). The treatments were added during OGD (OGD + GH group) and maintained during OGD/R (OGD/R + GH group). Likewise, cultures under normoxic conditions were also treated with GH (normoxia + GH group). As negative controls, each of the groups was incubated without GH.

### 2.4. Determination of Cell Survival

The effects of treatments upon cell survival were assessed by the MTT (3-[4,5-dimethilthiazol-2-yl]-2,5-diphenyltetrazolium bromide) assay, using a Vybrant kit (Molecular Probes, Eugene, OR, USA). Briefly, cells (1 × 10^5^) were cultured in 96-well plates (Costar Corning, NY, USA) and stabilized for 5 days at 39°C in a humidified chamber with an atmosphere of 95% air/5% CO_2_. After the treatments, culture media was substituted with DMEM medium without phenol red (Gibco, Grand Island, NY, USA), and the MTT reagent was added to each well (final concentration of 0.5 mg/mL) and then incubated for 4 h in a humidified atmosphere at 39°C. The resulting formazan crystals were dissolved in 0.1 mL of solubilization solution (1 g/mL SDS in 1 N HCl). Finally, the products were spectrophotometrically quantified at 570 nm, in a microplate reader (Bio-Rad, Hercules, CA, USA).

### 2.5. Immunocytochemistry

For immunocytochemical analysis, the cells (1 × 10^6^) were grown in 35 mm FluoroDish tissue culture dishes (World Precision Instruments Inc., Sarasota, FL, USA) following the methodology previously described. After the treatments, cell cultures were fixed with 4% paraformaldehyde in tris-buffered saline (TBS), pH 7.4, for 20 min, and then washed with TTBS (TBS + 0.05% Tween 20), 3 × 10 min. To block nonspecific staining, cells were incubated with 5% nonfat milk (Bio-Rad, Hercules, CA, USA) in TBS for 2 h at room temperature (RT) and then washed in TTBS (3 × 10 min). Primary antibodies (Table 1) were diluted (1 : 250) in 1% nonfat milk TTBS solution, added to the fixed cells, and then incubated overnight at RT. The next day, cells were washed in TTBS (3 × 10 min) and further incubated with the corresponding secondary antibodies (Table 1), for 2 h at RT, and then washed in TTBS (3 × 10 min). Later, the cells were incubated and counterstained with a 300 nM 4′6-diamidino-2-phenylindole solution (DAPI, Invitrogen) in TBS for 10 min and washed (3 × 10 min). The plates were analyzed using an Olympus BX51 fluorescence microscope, employing a ×20 objective. The total number of DAPI positive cells (DAPI+) was estimated in at least 20 fields, in two different dishes per experiment, with a total of three independent experiments. Image analysis was performed using FIJI-ImageJ software (https://fiji.sc).

### 2.6. SDS-PAGE and Western Blot Analysis

Hippocampal cell cultures (4 wells per experimental group) were homogenized with an ultrasonicator (GE 130PB, Cole-Parmer, Vermon Hills, IL, USA) in 0.05 M HCl-Tris, pH 9.0 buffer, in the presence of a protease inhibitor cocktail (Mini-complete, Roche, Mannheim, Germany). The extracts were centrifuged (12,500 rpm, 15 min), and the supernatants were collected. Total proteins were determined by the Bradford assay (Bio-Rad, Hercules, CA, USA), and 50 *μ*g of protein/lane was separated in 12.5% SDS-PAGE slabs and then transferred onto nitrocellulose membranes (Bio-Rad, Hercules, CA, USA) as previously described [24]. For immunoblotting, membranes were blocked with 5% nonfat milk (Bio-Rad) in TBS for 2 h. Then, membranes were incubated overnight at RT with the corresponding primary antibodies (Table 1) in TTBS. After washing the membranes with TTBS (3 × 10 min), they were incubated for 2 h with the corresponding HRP-conjugated secondary antibodies. Immunoreactive bands were visualized using the ECL blotting detection reagent (Amersham-Pharmacia, Buckinghamshire, UK) on autoradiography film (Fujifilm, Tokyo, Japan). Kaleidoscope molecular weight markers (Bio-Rad) were used as reference for molecular mass determination.

### 2.7. Reverse Transcription Polymerase Chain Reaction (RT-PCR)

Total RNA was extracted from cells in each well (4 wells per experimental condition) by adding 100 *μ*L of TRIzol (Invitrogen, Waltham, MA, USA) according to the manufacturer's indications. RNA was purified from cellular lysates using the Zymo Direct-zol purification kit (Zymo Research Corp., Irvine, CA, USA). Genomic DNA contamination was removed by DNAse I treatment (Invitrogen, Waltham, MA, USA) for 15 min at RT. The cDNA was synthetized from 2 *μ*g of total RNA using 100 U of M-MLV reverse transcriptase (Promega, Madison, WI, USA), 1 mM dNTPs, 0.5 *μ*g oligo d(T), and 0.5 *μ*g random hexamers, for 60 min at 42°C, in a final volume of 40 *μ*L.

### 2.8. Quantitative PCR (qPCR)

The expression of target genes (Table 2) was quantified by qPCR using an ABI-PRISM 7900HT system (Applied Biosystems, Foster, CA, USA), using SYBR green (Maxima, Thermo Scientific, Waltham, MA, USA) in 10 *μ*L final volume containing 3 *μ*L of diluted cDNA and 0.5 *μ*L of each specific primer (0.5 *μ*M). Primer sets used (Table 2) were designed to amplify avian mRNAs and to cross intron-exon boundaries to control for genomic DNA contamination. Reactions were performed under the following conditions: initial denaturation at 95°C for 10 min, followed by 40 cycles of 95°C for 15 sec, 60°C for 30 sec, and 72°C for 30 sec. Dissociation curves were included after each qPCR experiment to ensure primer specificity. The relative abundance of the corresponding mRNAs was calculated using the comparative threshold cycle (Ct) method, employing the formula 2^-∆∆CT^ [35–37]. Gene expression determinations were performed in duplicate, from 3-4 independent experiments.

### 2.9. Determination of Apoptosis by Caspase-3 Activity

Apoptotic cell death in cell cultures was analyzed by using a caspase-3 colorimetric assay kit (Assay Designs Inc., Ann Arbor, MI, USA). The samples (8 *μ*g protein) of cell lysates from each treatment, standards, p-nitroaniline (pNA) standard, and blank controls were placed in 96-well microplates, by duplicate. After a 3 h incubation at 37°C, the reaction was stopped with 1 N HCl, and absorbance at 405 nm was read immediately in a 3350-UV microplate reader (Bio-Rad, Hercules, CA, USA). The caspase-3 activity was calculated as units per microgram of protein [38], normalized and expressed as percent activity relative to the OGD condition (which was considered as 100%).

### 2.10. Neurite Growth Analysis

After the treatments, cells were stained with the neuronal biomarker *β*-III-tubulin and visualized using an Olympus BX51 fluorescence microscope with a ×40 objective. Images of individual neurons were captured with Lumenera software (Ottawa, CA). Neurites of each neuron (including all of its ramifications) were traced using the Simple Neuro Tracer (Fiji freeware [39]), and total dendritic length and branch order analysis were automatically measured. For each group, 20 neurons from 8-10 randomly selected fields were analyzed, from three independent experiments.

### 2.11. Statistical Analysis

In all experiments, values were expressed as mean ± SEM. Significant differences between groups or treatments were determined by either one-way ANOVA or two-way ANOVA and Tukey's posthoc test. Unpaired Student's *t*-test was used to compare between two groups where appropriate. Values of *p* < 0.05 were considered to be statistically significant.

## 3. Results

### 3.1. Characterization of Hippocampal Primary Cell Cultures and Effect of OGD Injury upon Cell Survival

**Figure 1:**
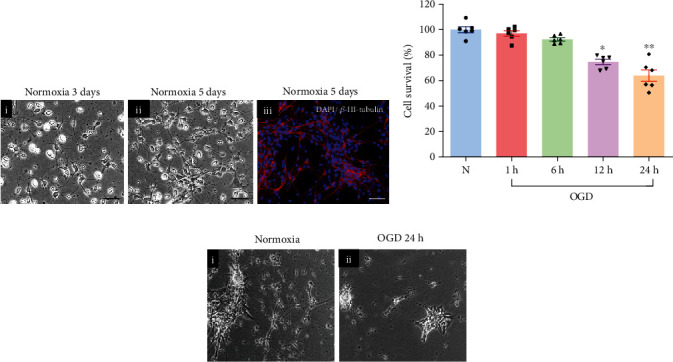
Characterization of hippocampal primary cell cultures and effects of OGD injury. (a) Typical morphology of healthy hippocampal primary cell cultures from chick embryos under normoxic conditions. (i) At day 3, neurons were small with round cell bodies and short neurites. (ii) At day 5, neurons showed longer neurites forming extensive neural networks. (iii) Merged immunofluorescence images from cultured neurons showing cell bodies stained with DAPI (blue) and neurites with *β*-III-tubulin (red). Scale bar: 50 *μ*m. (b) Time course of cell survival during OGD incubation. Cell survival was evaluated by the MTT assay. (c) Representative image from hippocampal cell cultures exposed to either normoxic condition or OGD for 24 h. Scale bar: 10 *μ*m. Bars represent mean ± SEM. Asterisks (^∗^) indicate significant differences in comparison to the control (^∗^*p* < 0.05; ^∗∗^*p* < 0.01) as assessed by one-way ANOVA and Tukey as posthoc test.

The typical morphology of the cultured embryonic chicken hippocampal neurons used in this study is shown in Figure 1, under normoxic conditions (Nx). After three days in culture, round cell bodies with short neurites were observed in the phase contrast microscope (Figure 1(a)i). At day 5, an increase in neurite length and arborization was clearly noticeable (Figure 1(a)ii). Over 80% of total cultured cells at this stage were neurons expressing *β*-III-tubulin (Figure 1(a)iii) and DCX (Figure S1) immunoreactivities (IR). In addition, other cellular types, such as GFAP-IR cells, were also observed in the cultures, although in a lower proportion (Figure S1).

To characterize the effect of hypoxia-ischemia upon cell viability, hippocampal neuron cultures were exposed to OGD incubation conditions for 1, 6, 12, or 24 h. As shown in Figure 1(b), there was a progressive, time-dependent, deleterious effect on the viability of neurons, which was significantly reduced at 12 h and 24 h (down to 71.52 ± 5.11% and 64 ± 5.5%, respectively), in comparison to the normoxic conditions (100% at the beginning and 98.2 ± 3.75% after 24 h of incubation). Since the neuronal injury was more evident after exposure to OGD for 24 h (Figure 1(c)), this was the time frame used in the rest of the experiments in the study.

### 3.2. Effects of Reoxygenation (OGD/R) on OGD Injured Neurons

**Figure 2:**
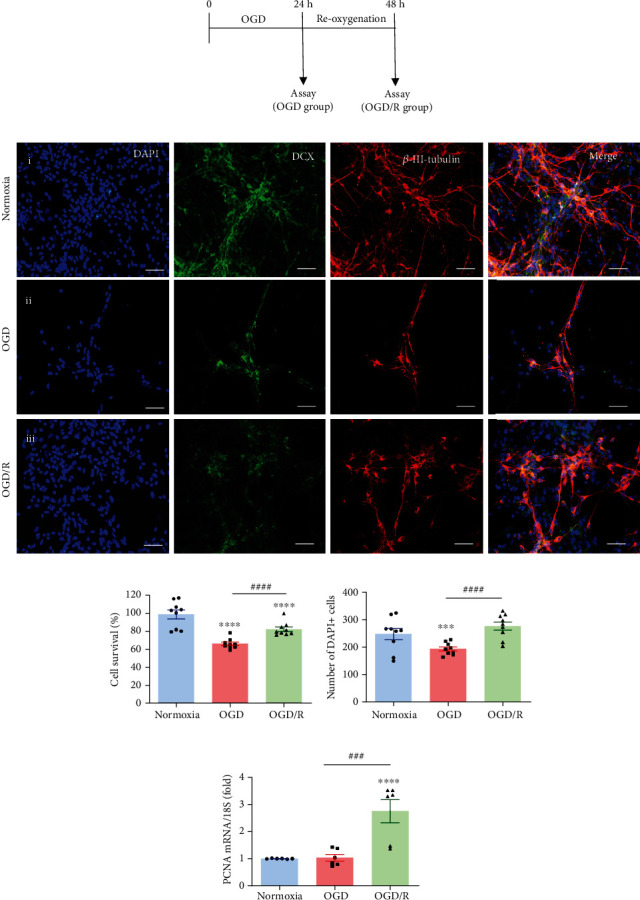
Effects of OGD and reoxygenation (OGD/R) in hippocampal cell cultures. **(**a) Time course schematic representation of OGD and reoxygenation (OGD/R) experimental conditions. (b) Representative immunofluorescence images showing effects of incubation conditions upon cultured hippocampal cells exposed to (i) normoxia, (ii) OGD, or (iii) OGD/R. Cell nuclei were stained with DAPI (blue) and neurons with anti-DCX (green) and anti-*β*-III-tubulin (red), and the images were merged. Scale bar: 50 *μ*m. (c) Cell survival was evaluated by the MTT assay in each experimental group. (d) Quantification of cell nuclei stained with DAPI from each experimental group. (e) The expression of PCNA mRNA was analyzed by RT-qPCR. Relative mRNA expression values were corrected by the comparative threshold cycle (Ct) method and employing the formula 2^−*ΔΔ*CT^. Ribosomal 18S RNA was used as housekeeping gene. Bars represent mean ± SEM. Asterisks (^∗^) indicate significant differences in comparison to control (^∗∗∗^*p* < 0.001; ^∗∗∗∗^*p* < 0.0001), and number sign (#) shows differences between experimental groups (*###p* < 0.001; *####p* < 0.0001) as assessed by one-way ANOVA and Tukey as posthoc test.

In order to mimic the reperfusion phase after hypoxia-ischemia in our in vitro injury model, hippocampal cell cultures were first exposed to OGD for 24 h, as mentioned above, and then incubated under OGD/R conditions for another period of 24 h (Figure 2(a)). The neurons were immunostained with antibodies against DCX and *β*-III-tubulin, and cell nuclei were stained with DAPI (Figure 2(b)). The immunofluorescence image analysis showed that exposition to OGD injury provoked a drastic reduction in the number of cells (DAPI+), as well as in the morphology of DCX-IR and *β*-III-tubulin-IR neurons (Figure 2(b)ii) in comparison with cultures incubated under Nx conditions (Figure 2(b)i). Interestingly, an evident recovery of cell number and morphology of the DCX-IR and *β*-III-tubulin-IR neurons was observed after the OGD/R phase (Figure 2(b)iii).  Figure 2(c) shows that in comparison with the normoxic cultures, cell survival (100 ± 3.58%), as determined by the MTT assay, was significantly reduced (down to 63.88 ± 0.58%, *p* < 0.001) in the OGD group, whereas reoxygenation (OGD/R group) induced a partial increase of viable cells (82.31 ± 1.57%, *p* < 0.0001) in relation to the injured group; although, it was still different than the Nx group.

As mentioned above, the number of DAPI+ cells was strongly affected by the treatments.  Figure 2(d) shows that, in comparison with the Nx group (248 ± 9.5 cells/field), the cultures exposed to OGD injury showed a strong reduction (191.3 ± 8.37 cells/field, *p* < 0.001); however, a significant increase in the DAPI+ cell number was observed in the OGD/R group (274.8 ± 8.42 cells/field, *p* < 0.001), which recovered levels similar to the Nx conditions. To determine if this effect was due to an increase in cell proliferation, the expression of PCNA mRNA was evaluated by qPCR (Figure 2(e)), and results showed a very significant increase (3.3-fold, *p* < 0.001) in the OGD/R group, in comparison to both Nx and OGD groups.

### 3.3. Reoxygenation (OGD/R) Induces Neurite Outgrowth and the Expression of Synaptogenic Markers

**Figure 3:**
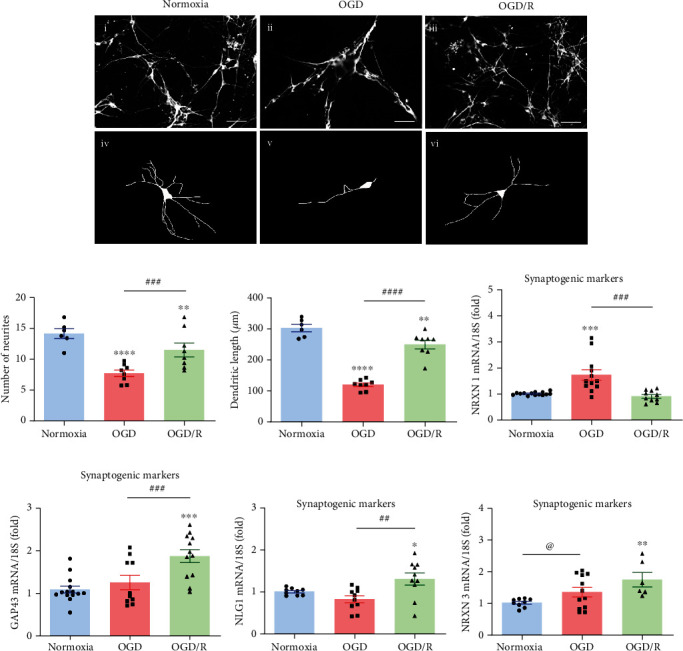
Effects of OGD and reoxygenation (OGD/R) on neurite outgrowth and expression of synaptogenic markers. (a) Representative microphotographs of hippocampal cultures in each experimental group are shown. (i-iii) Neurons stained with *β*-III-tubulin. Scale bar: 50 *μ*m. (iv-vi) Schematic drawings from *β*-III-tubulin-IR neurons used for morphometric analysis. (b) The number of neurites was evaluated in 10 individual neurons for each experimental group. (c) For dendritic length analysis, individual neurons were selected and drawn using the Simple Neuro Tracer software. Lower panels show the relative expression of (d) NRXN 1, (e) NRXN 3, (f) NLG1, and (g) GAP43 mRNAs, as determined by RT-qPCR. Relative mRNA expression values were corrected by the comparative threshold cycle (Ct) method and employing the formula 2^−*ΔΔ*CT^. Ribosomal 18S RNA was used as housekeeping gene. Bars represent mean ± SEM. Asterisks (^∗^) indicate significant differences in comparison to control (^∗^*p* < 0.05; ^∗∗^*p* < 0.01; ^∗∗∗^*p* < 0.001; ^∗∗∗∗^*p* < 0.0001), and number sign (#) shows differences between experimental groups (#*p* < 0.05; ##*p* < 0.01; ###*p* < 0.001; ####*p* < 0.0001) as assessed by one-way ANOVA and Tukey as posthoc test. An unpaired Student's *t*-test was used to compare normoxia versus OGD (g), and the at sign (@) indicates a significant difference (@,*p* < 0.05).

Figure 3(a) shows the morphological analysis of *β*-III-tubulin-IR hippocampal neurons with an epifluorescence microscope, to determine the effect of each treatment condition on the number of neurites, length, and branching. In comparison with the control Nx group (14.11 ± 0.8, Figures 3(a)i, 3(a)iv, and 3(b)), the number of neurites was significantly decreased in the OGD group to around one half (7.84 ± 0.45, *p* < 0.05, Figures 3(a)ii, 3(a)v, and 3(b)), whereas the average length of the longest neurites was markedly reduced, up to 60% (300.5 ± 17.95 *μ*m vs. 121 ± 5.2 *μ*m, respectively; *p* < 0.0001, Figures 3(a)ii, 3(a)v, and 3(c)). Interestingly, reoxygenation of the hippocampal cultures induced a partial recovery of these parameters, in comparison to the OGD group, although did not reach those in the Nx group. Thus, the number of neurites was significantly increased (11.22 ± 0.8, *p* < 0.001, Figures 3(a)iii, 3(a)vi, and 3(b)), and the average length was also augmented (240.9 ± 11.43 *μ*m, *p* < 0.0001, Figures 3(a)iii, 3(a)vi, and 3(c)) in the OGD/R group.

The expression of several specific pre and postsynaptic markers in response to the different treatments was also studied by qPCR. It was found that the expression of NRXN1 and NRXN3 mRNAs was significantly increased (1.7-fold, *p* < 0.001, Figure 3(d); 1.3-fold, *p* < 0.05, Figure 3(e); respectively) under OGD conditions in comparison to the Nx group, but the NRXN3 expression returned to similar control levels after the OGD/R stage. On the other hand, the expression of NRXN3, NLG1, and GAP43 mRNAs was no different from the normoxic controls when the cultures were exposed to OGD injury but were significantly increased after OGD/R (1.7-fold, *p* < 0.001, Figure 3(e); 1.3-fold, *p* < 0.05, Figure 3(f); and 1.8-fold, *p* < 0.001, Figure 3(g); respectively).

### 3.4. Effects of OGD Injury and Reoxygenation (OGD/R) upon mRNA Expression of Local Neurotrophins and Growth Factors

**Figure 4:**
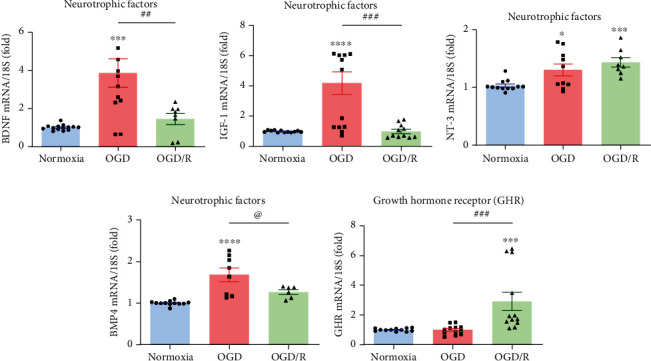
Effect of OGD and reoxygenation (OGD/R) upon BDNF, IGF-1, NT-3, BMP4, and GHR mRNA expression. Panels show the relative expression of (a) BDNF, (b) IGF-1, (c) NT-3, (d) BMP4, and (e) GHR mRNAs, determined by RT-qPCR. Relative mRNA expression values were corrected by the comparative threshold cycle (Ct) method and employing the formula 2^−*ΔΔ*CT^. Ribosomal 18S RNA was used as housekeeping gene. Bars represent mean ± SEM. Asterisks (^∗^) indicate significant differences in comparison to control (^∗^*p* < 0.05; ^∗∗∗^*p* < 0.001; ^∗∗∗∗^*p* < 0.0001), and number sign (#) shows differences between experimental groups (#*p* < 0.05; ##*p* < 0.01; ###*p* < 0.001) as assessed by one-way ANOVA and Tukey as posthoc test. An unpaired Student's *t*-test was used to compare OGD versus OGD/R (d), and the at sign (@) indicates a significant difference (@*p* < 0.05).

The expression of several endogenous neurotrophins and growth factors in response to the different treatments was analyzed by qPCR. Results showed that, in comparison to the Nx controls, the exposure of hippocampal cultures to OGD injury significantly increased the mRNA expression of BDNF (3.8-fold, *p* < 0.0001, Figure 4(a)), IGF-1 (4.1-fold, *p* < 0.0001, Figure 4(b)), and BMP4 (1.6-fold, *p* < 0.001, Figure 4(d)). Interestingly, reoxygenation of the cultures restored the expression of these markers to similar levels as in Nx controls (Figures 4(a), 4(b), and 4(d)). On the other hand, as shown in Figure 4(c), the expression of NT-3 mRNA was significantly increased in both OGD (1.3-fold, *p* < 0.001) and OGD/R (1.4-fold, *p* < 0.0001) groups, respectively, in comparison to the Nx control group. Furthermore, the expression of GHR mRNA was strongly upregulated (2.9-fold, *p* < 0.001) only in the OGD/R group, in comparison to the Nx and OGD groups (Figure 4(e)).

### 3.5. Neuroprotective Effects of GH in Hippocampal Cell Cultures Exposed to OGD and Reoxygenation (OGD/R)

**Figure 5:**
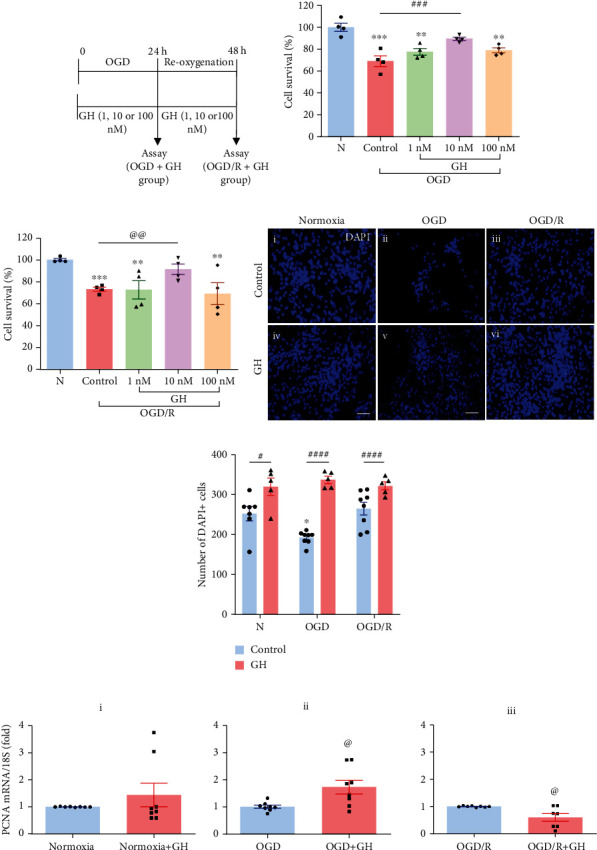
Neuroprotective effects of growth hormone (GH) in hippocampal cell cultures exposed to OGD and reoxygenation (OGD/R) conditions. (a) Treatments and time course schematic representation of OGD and reoxygenation (OGD/R) protocols in the experimental groups. Cell survival was evaluated by the MTT assay in primary cultures treated with different doses (1, 10, 100 nM) of GH and exposed to (b) OGD and (c) OGD/R incubation conditions. Bars represent mean ± SEM. Asterisks (^∗^) indicate significant differences in comparison to control (^∗∗^*p* < 0.01; ^∗∗∗^*p* < 0.001), and number sign (#) shows differences between experimental groups (###, *p* < 0.001) as assessed by one-way ANOVA and Tukey as posthoc test. An unpaired Student's *t*-test was used to compare control versus 10 nM GH (c), and the at sign (@) indicates a significant difference (@@*p* < 0.01). (d) Representative immunofluorescence images from each experimental group: (i) normoxia, (ii) OGD, (iii) OGD/R, (iv) normoxia + GH, (v) OGD + GH, and (vi) OGD/R + GH. Cell nuclei were stained with DAPI. Scale bar: 50 *μ*m. (e) Quantification of the number of cells stained with DAPI in each experimental group. Bars represent mean ± SEM. Asterisks (^∗^) indicate significant differences in comparison to control (^∗^*p* < 0.05), and number sign (#) shows differences between experimental groups (#*p* < 0.05; ####*p* < 0.0001) as assessed by two-way ANOVA and Tukey as posthoc test. (f) Expression of PCNA mRNA, as determined by RT-qPCR in each experimental group: (i) normoxia, (ii) OGD, and (iii) OGD/R. Relative mRNA expression values were corrected by the comparative threshold cycle (Ct) method and employing the formula 2^−*ΔΔ*CT^. Ribosomal 18S RNA was used as housekeeping gene. Bars represent mean ± SEM. The at sign (@) indicates a significant difference in comparison to control (@*p* < 0.05) as assessed by unpaired Student's *t*-test.

Figure 5(a) shows the protocol designed to determine the protective effect of GH treatment when coadministered simultaneously during OGD conditions for 24 h or during reoxygenation (OGD/R) conditions for additional 24 h, upon cell survival (MTT assay), cell number (DAPI+ nuclei), and proliferation (PCNA mRNA expression). As described before, in comparison to Nx conditions (100 ± 3.7%), OGD-injury conditions provoked a marked reduction of cell survival (down to 69.0 ± 4.87%, *p* < 0.001); however, the administration of GH (1, 10, or 100 nM) induced a dose-dependent response that partially improved cell viability (77.62 ± 3.05%, 89.5 ± 1.48%, and 79.01 ± 2.34%, respectively). Interestingly, with the 10 nM GH dose, there was no statistical difference between the Nx group and the OGD + GH group (Figure 5(b)). On the other hand, Figure 5(c) shows that the cell viability was significantly reduced to 73.3 ± 3% under OGD/R in comparison to Nx group, and the GH treatment stimulated the recuperation of cell survival only at the 10 nM dose (91.61 ± 4.3%, *p* < 0.01). Therefore, this was the dose selected to use in the rest of the experiments.

As shown in Figure 5(d), the administration of 10nM GH to hippocampal cultures stimulated an increase in the cell number, on either incubation condition, as compared with the corresponding controls.  Figure 5(e) shows the quantitation of DAPI+ cells in each experimental condition; as mentioned before, in comparison to the Nx group, OGD injury provoked a reduction of 23.7% in the number of cells (248.9 ± 10 vs. 189.4 ± 7 cells/field, respectively), but reoxygenation (OGD/R group) significantly reversed this effect (267.2 ± 7 cells/field), stimulating a 41.1% increase in the number of DAPI+ cells in comparison to the OGD condition. The addition of 10 nM GH significantly augmented the number of cells in all the cultures compared with the untreated controls: 30.9% in the normoxic group (248.9 ± 10 vs. 325.8 ± 8 cells/field, *p* < 0.05); 78.9% in the OGD group (189.4 ± 4 vs. 338.8 ± 15 cells/field, *p* < 0.0001) and 28.3% in the OGD/R group (267.2 ± 7 vs. 342.9 ± 14 cells/field, *p* < 0.0001), respectively. Remarkably, the strongest neuroprotective effect of GH was observed in the OGD injured group (Figure 5(e)).

Figure 5(f) shows the effects of 10 nM GH treatment upon the PCNA mRNA expression in each of the experimental conditions; under normoxic environment, there was no significant differences between the GH-treated group and its nontreated control (1.43-fold, *p* = 0.27, Figure 5(f)i); however, the PCNA expression was appreciably increased when GH was added under OGD conditions (1.73-fold, *p* < 0.01, Figure 5(f)ii). In contrast, a significant reduction in the PCNA mRNA expression in the OGD/R+GH group (0.59-fold, *p* < 0.01, Figure 5(f)iii) was observed.

### 3.6. GH Reduces Apoptosis and Increases Doublecortin-IR in Hippocampal Cultures Exposed to OGD and Reoxygenation (OGD/R)

**Figure 6:**
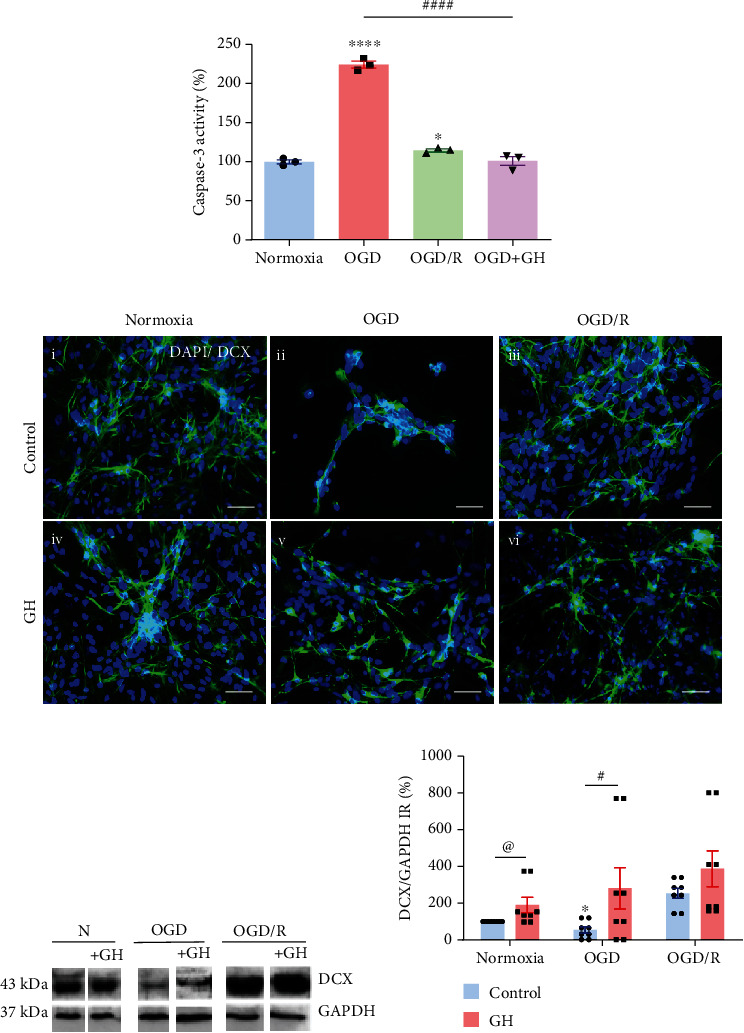
Effect of growth hormone (GH) upon apoptosis and doublecortin-immunoreactivity (DCX-IR) in hippocampal cultures exposed to OGD and reoxygenation (OGD/R) conditions. (a) Caspase-3 activity as determined with a colorimetric assay. Bars represent mean ± SEM. Asterisks (^∗^) indicate significant differences in comparison to control (^∗^*p* < 0.05; ^∗∗∗∗^*p* < 0.0001), and number sign (#) shows differences between experimental groups (####*p* < 0.0001) as assessed by one-way ANOVA and Tukey as posthoc test. (b) Representative immunofluorescence merged images from each experimental group: (i) normoxia, (ii) OGD, (iii) OGD/R, (iv) normoxia + GH, (v) OGD + GH, and (vi) OGD/R + GH. Cell nuclei were stained with DAPI (blue) and neurons with anti-DCX (green). Scale bar: 50 *μ*m. (c) Representative Western blots for DCX-IR. (d) Densitometric analysis of immunoblots for DCX-IR (corrected and normalized with GAPDH immunoreactivity). Bars represent mean ± SEM. Asterisks (^∗^) indicate significant differences in comparison to control (^∗^*p* < 0.05), and number sign (#) shows differences between experimental groups (#*p* < 0.05) as assessed by two-way ANOVA and Tukey as posthoc test. An unpaired Student's *t*-test was used to compare control versus GH in Nx condition (d), and the at sign (@) indicates a significant difference (@*p* < 0.05).

Figure 6(a) shows that exposure of cells to OGD injury provoked a very significant increase (224.1 ± 4.3%, *p* < 0.0001) in apoptotic death, as determined by caspase-3 activity, in comparison to the Nx group (100 ± 2.5%), while reoxygenation conditions (OGD/R) mostly reversed this deleterious effect (114.2 ± 1.9%). Also, treatment of OGD injured cells with 10 nM GH effectively decreased caspase-3 activity, returning its levels to those observed in normoxic conditions (100.7 ± 5.8%).

Immunofluorescence analysis in Figure 6(b) shows the effects of the different experimental conditions upon the number of DAPI+ nuclei and the presence of DCX-IR cells. As expected, cultures exposed to OGD harm suffered a drastic reduction of DCX-IR neurons (Figure 6(b)ii); however, reoxygenation conditions (OGD/R) provoked a recovery of the cultures (Figure 6(b)iii). Interestingly, GH treatment to the OGD group also annulled the noxious effect and significantly increased the number of DAPI+ and DCX-IR cells (Figure 6(b)v). On the other hand, addition of GH under normoxic or OGD/R conditions did not show any evident changes, as compared with their respective controls.

The effects of GH during OGD and OGD/R on DCX-IR were confirmed by Western blot analysis (Figures 6(c) and 6(d)). Results showed that, in comparison to normoxic conditions (100%), OGD injury significantly reduced DCX-IR (down to 72.51 ± 25.6%, *p* < 0.05), while reoxygenation induced a drastic increase (up to 252.8 ± 41.6%, *p* < 0.05). On the other hand, administration of GH increased DCX-IR in all conditions, but it was particularly high when added to Nx condition (220 ± 48.66%, *p* < 0.05) and OGD injured cells (375 ± 127.9%, *p* < 0.05).

### 3.7. GH Protects and Induces Neurite Outgrowth in Cultures Exposed to OGD and Reoxygenation (OGD/R)

**Figure 7:**
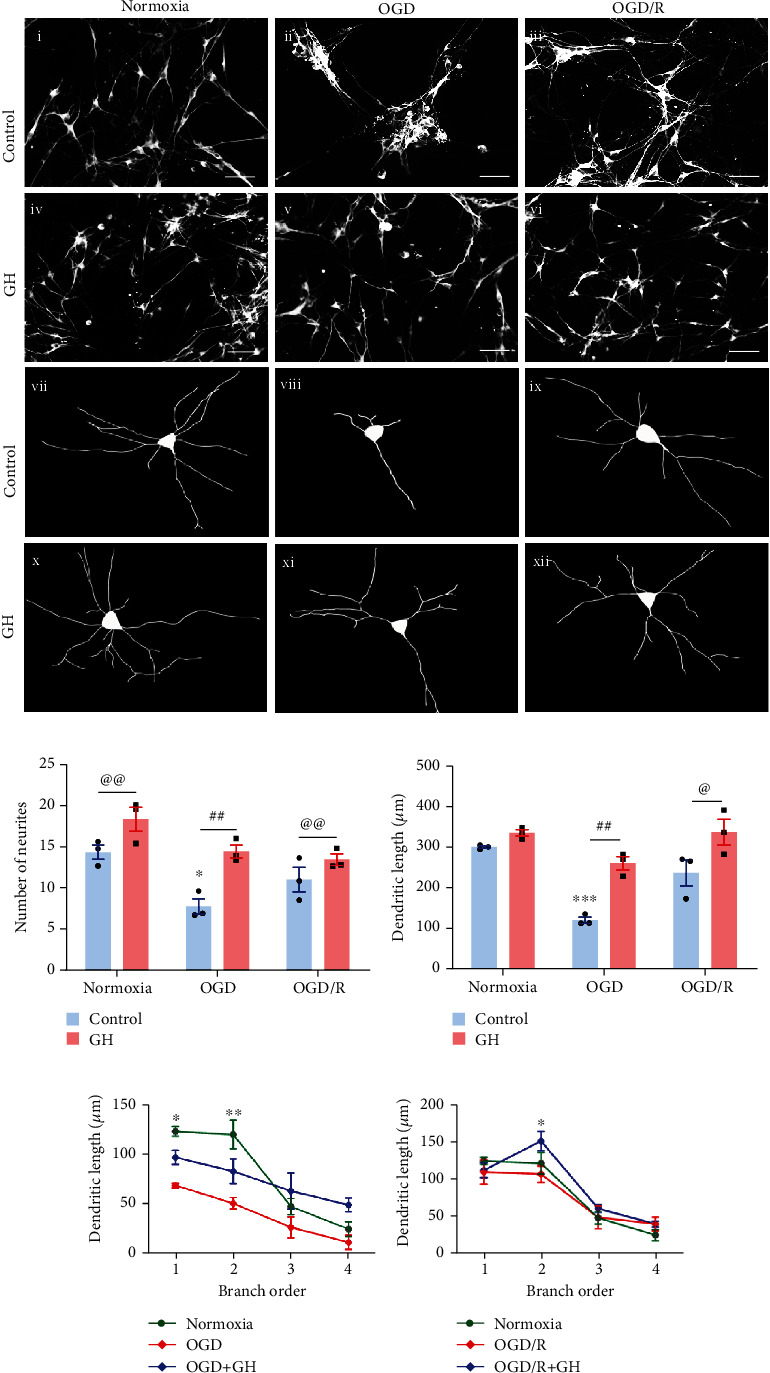
Growth hormone (GH) increases the number of neurites and dendritic length after OGD and reoxygenation (OGD/R) conditions. (a) Representative microphotographs of hippocampal cultures in each experimental group are shown. (a–f) Neurons stained with *β*-III-tubulin in: (i) normoxia, (ii) OGD, (iii) OGD/R, (iv) normoxia + GH, (v) OGD + GH, and (vi) OGD/R + GH. Scale bar: 50 *μ*m. (vii-xii) Schematic drawings from neurons stained with *β*-III-tubulin used for morphometric analysis, in (vii) normoxia, (viii) OGD, (ix) OGD/R, (x) normoxia + GH, (xi) OGD + GH, and (xii) OGD/R + GH. (b) The number of neurites was evaluated in 10 individual neurons for each group. (c) For dendritic length analysis, individual neurons were selected and drawn using the Simple Neuro Tracer software. Lower panels show the dendritic length by branch order analysis of hippocampal neurons exposed to (d) OGD and (e) OGD/R conditions. Bars represent mean ± SEM. Asterisks (^∗^) indicate significant differences in comparison to control (^∗^*p* < 0.05; ^∗∗^*p* < 0.01; ^∗∗∗^*p* < 0.001), and number sign (#) shows differences between experimental groups (#*p* < 0.05; ##*p* < 0.01) as assessed by two-way ANOVA and Tukey as posthoc test. An unpaired Student's *t*-test was used to compare control versus GH in normoxia condition and control versus GH in OGD/R condition (panel b), and control versus GH in OGD/R condition (c), and the at sign (@) indicates significant differences (@*p* < 0.05; @@*p* < 0.01).

Figure 7(a) shows the morphometric analysis performed in *β*-III-tubulin-IR cells present in the hippocampal cultures exposed to the different experimental conditions and treatments. It was clear that OGD injury provoked a drastic reduction in the number of immunoreactive cells and the number and length of neuronal projections (Figures 7(a)ii and 7(a)viii). These harmful effects in the OGD group were reversed by either exposing the cells to reoxygenation (Figure 7(a)ix) or by treating them with GH (Figure 7(a)xi).  Figure 7(b) shows the corresponding quantitative analysis in each condition; in comparison to the Nx group (14.11 ± 0.8), the number of neurites was significantly reduced to almost half in the OGD group (7.84 ± 0.45, *p* < 0.01), whereas reoxygenation induced a partial recovery (11.22 ± 0.8) in the OGD/R group, increasing the number of neurites (43.1%) in relation to the injured group, although did not reach the Nx levels. On the other hand, GH treatment provoked increases in the number of neurites in all three conditions in comparison with their respective untreated controls: Nx, 30.3%; OGD, 87.8%; and OGD/R, 19.7%, respectively. The most striking effect, by far, was observed in the OGD + GH group, where GH treatment practically reestablished the levels obtained in the Nx control (14.73 ± 0.8 vs. 14.11 ± 0.8, respectively) and almost doubled those in the injured group control (7.84 ± 0.45). Consistent with these results, Figure 7(c) shows that the dendritic length was also drastically reduced in the OGD group in comparison with the Nx control (300.5 ± 17.95 vs. 121.7 ± 5.2 *μ*m, *p* < 0.001), and reoxygenation was capable to induce a recovery (240 ± 11.4 *μ*m). Remarkably, GH treatment stimulated a significant increase (119.4%) of dendritic length in the OGD + GH group in comparison to the untreated OGD group (267 ± 14.6 vs. 121.7 ± 5.2 *μ*m, *p* < 0.001, respectively), as well as in the OGD/R + GH group in relation to its control (295.9 ± 15.22 vs. 240.9 ± 11.42 *μ*m, *p* < 0.05, respectively). The branch-order analysis revealed significant increases in the length of dendrites of the first and second order (*p* < 0.001 and *p* < 0.05, respectively; Figure 7(d)), with no differences in the length of the third and fourth orders in the OGD + GH group. Similarly, OGD/R + GH group showed a significant increase in dendrites of the second order (Figure 7(e)).

### 3.8. GH Promotes Synaptogenic Marker Expression in Response to OGD and Reoxygenation (OGD/R)

**Figure 8:**
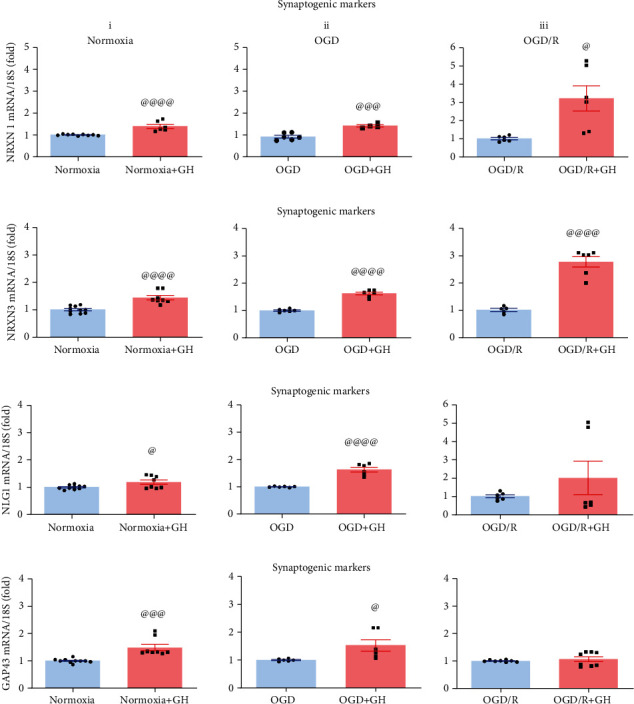
Growth hormone (GH) increases the expression of synaptogenic marker mRNAs in hippocampal cultures exposed to OGD and reoxygenation (OGD/R) conditions. Panels show the relative expression of (a) NRXN 1, (b) NRXN 3, (c) NLG1, and (d) GAP43 mRNAs, in each group: (i) normoxia, (ii) OGD, and (ii) OGD/R, as determined by RT-qPCR. Relative mRNA expression values were corrected by the comparative threshold cycle (Ct) method and employing the formula 2^−*ΔΔ*CT^. Ribosomal 18S RNA was used as housekeeping gene. Bars represent mean ± SEM. The at sign (@) indicates significant differences in comparison to control (@*p* < 0.05; @@@*p* < 0.001; @@@@, *p* < 0.0001) assessed by unpaired Student's *t*-test.

The synaptogenic effect of GH on hippocampal cell cultures was analyzed by determining the expression of several markers by qPCR (Figure 8). It was found that NRX1 (Figure 8(a)) was significantly increased when GH was administered to the cultures under the three experimental conditions: Nx + GH (1.4-fold, *p* < 0.0001), OGD + GH (1.4-fold, *p* < 0.0006), and OGD/R + GH (3.2-fold, *p* < 0.03), compared with their respective untreated control groups. Similarly, the NRX3 mRNA expression (Figure 8(b)) was significantly increased in Nx + GH (1.5-fold, *p* < 0.0001), OGD + GH (1.6-fold, *p* < 0.0001), and OGD/R + GH (2.7-fold, *p* < 0.0001) groups, in relation to their controls. On the other hand, NLG1 (Figure 8(c)) and GAP-43 (Figure 8(d)) were significantly upregulated in the Nx + GH (1.5-fold, *p* < 0.001 and 1.5-fold, *p* < 0.0003) and the OGD + GH (1.6-fold, *p* < 0.0001 and 1.5-fold, *p* < 0.02, respectively) groups, but not in the OGD/R + GH group, compared to the untreated controls.

### 3.9. GH Effects upon the Expression of BDNF, IGF-1, NT-3, and GHR mRNAs in Cultures Exposed to OGD and Reoxygenation (OGD/R)

**Figure 9:**
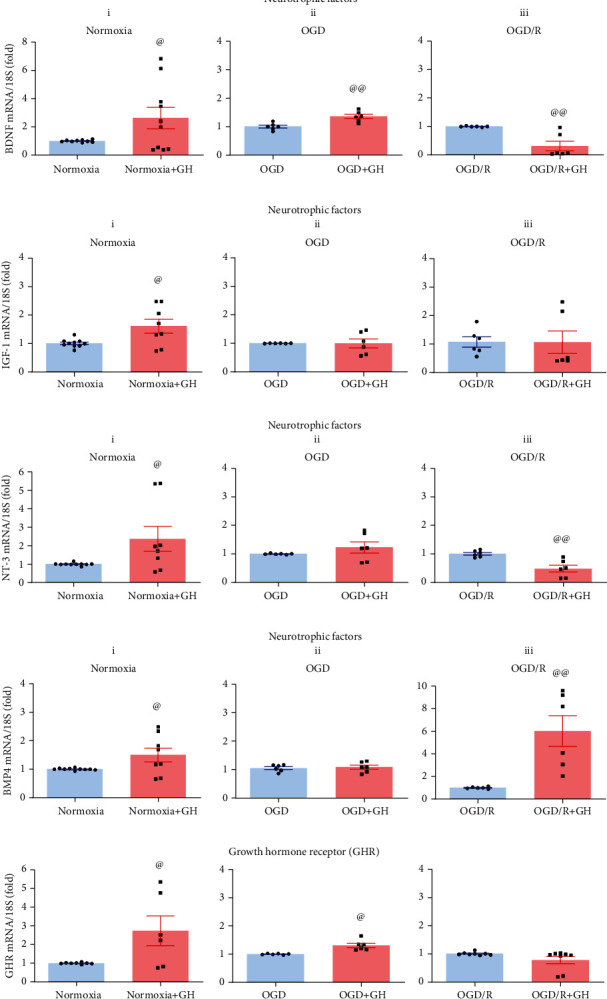
Effects of growth hormone (GH) upon BDNF, IGF-1, NT-3, BMP4, and GHR mRNA expression during OGD and reoxygenation (OGD/R) conditions. Panels show the relative expression of (a) BDNF, (b) IGF-1, (c) NT-3, (d) BMP4, and (e) GHR mRNAs, in each group: (i) normoxia, (ii) OGD, and (iii) OGD/R, as determined by RT-qPCR. Relative mRNA expression values were corrected by the comparative threshold cycle (Ct) method and employing the formula 2^−*ΔΔ*CT^. Ribosomal 18S RNA was used as housekeeping gene. Bars represent mean ± SEM. The at sign (@) indicates significant differences in comparison to control (@*p* < 0.05; @@*p* < 0.01) assessed by unpaired Student's *t*-test.

BDNF mRNA expression (Figure 9(a)) was significantly increased in Nx + GH (2.6-fold, *p* < 0.05) and OGD + GH (1.3-fold, *p* < 0.005) groups, but strongly reduced (down to 0.3-fold, *p* < 0.005) in the OGD/R + GH group, whereas the IGF-1 expression (Figure 9(b)) was appreciably increased (1.6-fold, *p* < 0.001) only in the Nx + GH group, in relation to their corresponding controls. On the other hand, the NT-3 mRNA expression (Figure 9(c)) was significantly increased in Nx + GH (2.3-fold, *p* < 0.003) and strongly reduced (down to 0.4-fold, *p* < 0.0005) in the OGD/R + GH group and showed no significant effect in the OGD + GH group. In turn, the BMP4 mRNA expression (Figure 9(d)) was substantially increased only in Nx + GH (1.5-fold, *p* < 0.05) and OGD/R + GH (5.9-fold, *p* < 0.01) groups. Lastly, the GHR mRNA expression (Figure 9(e)) was significantly upregulated in Nx + GH (2.7-fold, *p* < 0.002) and OGD + GH (1.3-fold, *p* < 0.002) groups, in relation to their respective untreated controls.

## 4. Discussion

This work aimed to study the neuroprotective role of GH in cultured embryonic chicken hippocampal neurons exposed to both oxygen-glucose deprivation (OGD) and reoxygenation (OGD/R) injuries. We demonstrated that reoxygenation of OGD injured hippocampal neurons activate endogenous mechanisms of neuroprotection and neuroplasticity that promote cell survival, neurite outgrowth, and synaptogenesis. We also demonstrated that neurotrophic factors such as BDNF, NT-3, IGF-1, and BMP4 are increased after an acute OGD damage. Interestingly, we observed that the GHR expression was upregulated during the reoxygenation stage. Furthermore, we found that treatment with GH enhances these neurorestorative processes by attenuating OGD-provoked neuronal apoptosis. Interestingly, GH treatment induced an increase on neurite outgrowth which positively correlated with an increase of the GAP43 expression and likely promoted synapse formation in primary hippocampal cultures after OGD injury, probably through a synergistic effect between GH and local neurotrophic factors.

In this work, we employed chicken embryonic primary hippocampal cell cultures. The rationale behind was that embryonic tissue is easier to dissociate, the risk of shearing damage to axons and dendrites is lower due to fewer adhesion contacts, isolation of a higher proportion of unharmed neurons is simpler, and the content of glial cells is smaller than in the adult brain tissue [40, 41]. Also, the prolonged survival and integrity of primary neuronal cultures from embryonic hippocampus are much easier to attain in comparison to mature hippocampal cultures, which present more difficulties [41]. These primary cultures are composed of several cell types, including hippocampal progenitor cells, mature neurons, and other nonneuronal cells such as oligodendrocytes and glia [40, 42, 43]. In our case, we cultured the embryonic primary neurons in serum-free Nb-B27 medium, which has been reported to improve neuronal survival and reduce growth of nonneuronal cells [40, 41, 43]. In effect, we found that, after 5 days in culture, over 80% of total cells were neurons expressing *β*-III-tubulin-IR and DCX-IR markers and, in a lower proportion, some glial cells expressing GFAP-immunoreactivity.

Cerebral ischemia is characterized by oxygen and glucose deprivation, commonly associated with a vascular obstruction in the brain blood circulation. When the blockage is removed, the tissue is reperfused, and oxygen and nutrients are restored, in some cases, enhancing tissue damage [44, 45]. In order to mimic a stroke injury, we used oxygen and glucose deprivation (OGD) and reoxygenation (OGD/R) of embryonic chick primary hippocampal cultures, as an in vitro cerebral ischemia-reperfusion model. Our results showed that the application of OGD during 24 h caused a severe damage in the neuronal cultures, by reducing cell survival to 64% in comparison with the normoxic conditions (98.2%) at the same incubation time. These data are consistent with findings reported in rat organotypic hippocampal slice cultures [46] and in ovo experiments where hypoxia for 24 h produced severe structural alterations in the hippocampus of chick embryos [47]. In contrast, we did not observe significant damage in OGD incubation during 1 or 6 h, as previously reported in murine primary cortical cultures [48], probably because hippocampal neurons are more resistant to hypoxic-ischemia insults [49]. Interestingly, when OGD injured neurons were reoxygenated for another 24 h, cell survival was significantly increased. These results contradict previous in vitro studies in mouse cortical and cerebellar cells reporting that reperfusion induced apoptosis and excitotoxic necrotic cell death by an overproduction of reactive oxygen species (ROS) [50, 51]. Recently, it was reported that reoxygenation decreased cell viability and increased necrosis in chicken cerebellar cell cultures [52]. Conversely, our results showed that hippocampal cell number was increased after the OGD/R phase. Furthermore, we observed that these conditions also increased the PCNA mRNA expression (3.3-fold), suggesting that reoxygenation activates proliferative responses that could influence the cell density. These findings are in accord with previous reports indicating that an ischemic insult promoted the proliferation of neural stem cells in the dentate gyrus of the rat hippocampus [17].

It has been described that after cerebral ischemia injury, neurite interconnections shrink and degenerate, thus losing the capacity to integrate neural networks [3]; although, it was shown that dendritic alteration in injured neurons can be reversed under certain conditions [53–56]. Previous studies performed in the visual cortex of the rat showed that, after damage, neurons may retain the capacity to survive, and injury-induced dendritic degeneration can be prevented, possibly through a mechanism known as axotomy, which consists of disconnecting the neurons that project to the site of injury, that activates a cascade of molecular and cellular events leading to the death of only the disconnected cells [55]. These results suggested that neurites are able to regenerate in functional neuronal networks and with partial recovery after neural harm [57]. Our results clearly showed that OGD significantly reduced the number and length of neurites in the hippocampal cultures. However, after the OGD/R phase, the morphometric parameters of those neurons were partially reestablished, probably due to the plastic capacity of neural cells to adapt to hypoxic conditions [58–60]. It is known that if the dendritic morphology is altered, then the synaptic capacity is also modified [61]; therefore, we evaluated the effects of OGD and OGD/R incubation conditions upon synaptic protection and regeneration.

Neurexins (NXRNs) and neuroligin-1 (NLG1) are synaptic cell-adhesion proteins that interact between pre and postsynaptic neurons, both are well-established markers of synaptic function in the hippocampus [62], and recently have been associated with neuroregeneration in damaged neuroretina [12]. Here, we found that NRXN3 and NLG1 mRNA expression showed no changes after OGD injury, as compared with the Nx conditions; however, after the OGD/R stage, their expression was significantly increased. Furthermore, GAP43, a synaptogenic marker associated to neurite outgrowth [63], was also increased after OGD/R. These data are consistent with our morphometric findings, in which we observed a significant increase in the number and length of neurites after the OGD/R phase. Taken together, our results suggest that OGD injured neurons activate endogenous mechanisms of neuroprotection and neuroplasticity that promote neurite outgrowth and functional synapsis formation during the OGD/R stage.

The mechanisms behind the brain capacity to promote neuroplasticity in response to neural lesions are still largely unknown. It has been reported that the expression and release of endogenous neurotrophic factors are altered by various experimental models of CNS injuries, including ischemic models [4, 25, 29]. After ischemic harm, the expression of growth factors such as BDNF, NT-3, and IGF-1 was increased [18–20]. These growth factors have been implicated in brain development, and their expression was increased after neural injury [64]. In this work, we found that the expression of BDNF, NT-3, and IGF-1 mRNAs was increased after OGD, and this clearly correlated with recovery effects observed after incubation in OGD/R conditions. It is known that BMP4 can act as a neurotrophic factor involved in the modulation of neurogenesis and plasticity in the hippocampus [65]. Also, a moderate although significant increase in the expression of BMP4 mRNA was observed in subcortical white matter following cerebral ischemia [66]. We noticed that the BMP4 mRNA expression was significantly increased after OGD injury, but returned to normoxic control levels after exposing the hippocampal cultures to OGD/R conditions. Our results insinuate that endogenous mechanisms of neuroprotection and neuroplasticity in response to an OGD injury could at least be partially mediated by the expression of neurotrophic factors (e.g., BDNF, NT-3, IGF-1, and BMP4).

It is known that GHR is expressed in several brain areas, including the hippocampus [67–69]. Moreover, it was described that the expression of GHR was strongly upregulated during recovery from focal hypoxic-ischemic brain injury, in the penumbra and infarcted regions of the cortex [22, 70]. In accordance with those results, here, we found a significant increase in the expression of GHR mRNA when the harmed cells were exposed to the reoxygenation phase, indicating that OGD injured neurons might be highly responsive to GH which, in turn, could induce restorative effects upon these neurons.

Also, GH can induce neuroprotection after cerebral stroke in humans [4–6] and in other animal models [4, 29, 71, 72]. After ischemic stroke, GH mRNA expression and GH content were increased in the rat hippocampus by chronic hypoxia [4], and treatment with exogenous GH effectively reduced hippocampal cell loss [22, 71, 73]. In accordance with those reports, our results demonstrated that GH was capable to significantly increase cell viability in OGD injured neurons. We found a dose-dependent response to GH administration upon cell viability, which exhibited a typical bell-shaped curve of GHR activation [74], and having an optimal effect at the 10 nM dose. The prosurvival actions of GH in response to neural lesions are well established; for example, GH treatment increased cell viability in primary chicken cerebellar cultures exposed to hypoxic-ischemic incubation conditions [24], as well as in avian and reptilian neuroretinal cells exposed to excitotoxic damage with kainate or glutamate [75–78], indicating a conserved neuroprotective action within the vertebrate CNS. Interestingly, the addition of GH during the OGD/R did not have a significant effect on cell viability; a possible explanation is that the apoptotic cell death process occurs during the first hours of OGD exposition, in accordance with previous reports [79], suggesting that neuroprotective actions of GH are required during the initial phase of injury.

GH is a potent mitogenic factor capable to exert a proliferative effect over striatal cells under normal conditions [8]. This mitogenic activity seemed to be important to protect hippocampal neurons against prolonged sleep loss [80]. Here, we observed that GH treatment during OGD and OGD/R conditions increased the number of cells in the cultures. This increase was apparently due to a proliferative effect of GH, since its presence promoted PCNA mRNA expression, thus indicating that growth hormone could have the potential to exert a direct mitogenic effect upon hippocampal cells in response to damage.

Doublecortin is a neural progenitor cell marker expressed in immature neurons during development and adult neurogenesis; it is associated with neuronal migration, axon growth, dendritic remodeling, and synapse maturation [81, 82]. We noticed the wide DCX expression in our embryonic hippocampal cultures, indicating that newly formed neurons were present. Our results showed that OGD significantly reduced the number of DCX+ cells while exposure to OGD/R conditions reversed those effects, probably due to the endogenous mechanisms of restoration mentioned previously. Recently, new actions for the DCX family have been associated to cell survival and regeneration [83]; therefore, it is possible that GH neuroprotective effects include DCX involvement, since we found that GH treatment during OGD injury significantly increased DCX-IR, to reach similar levels as in the normoxic controls. Likewise, GH addition during OGD/R maintained the effect observed during OGD harm. These results support the notion that GH may exert a neurotrophic action to promote neuron survival, in accordance with analogous reports in embryonic striatal neural precursor cells [8]. The mechanism by which GH could exert its neuroprotective effects could be associated with an antiapoptotic action reported previously for this hormone in the neural retina [7, 75, 78, 84], cerebellum [24, 52], and hippocampus [71]. Accordingly, we observed that caspase-3 activity was strongly increased during OGD injury, and then significantly reduced after the cells were exposed to OGD/R conditions. Interstingly, the addition of GH during ODG conditions provoked a striking reduction of caspase-3 activity, suggesting that prosurvival actions of GH in hippocampal neurons are exerted through an antiapoptotic mechanism.

Besides increasing apoptotic and necrotic cell death, the deleterious effects provoked by hypoxic-ischemic injury also included important morphological and synaptic changes, such as a reduction of neurite number and length. Beneficial effects of GH treatment upon structural and synaptic plasticity have been reported, either by increasing the dendritic length and number of dendritic spines in the cortex and hippocampus of the rat [10, 11], by regulating the expression of *α*-amino-3-hydroxyl-5-methyl-4-isoxazol-propionate (AMPA) and N-methyl-D-aspartate (NMDA) receptors, or by increasing the expression of postsynaptic density 95 protein [85]. Here, we demonstrated that GH treatment improved structural and synaptic plasticity in the injured hippocampal cell cultures. Thus, GH addition increased the number and length of primary neurites in OGD conditions, while under the OGD/R phase, it only augmented the length of neurites of the second order of branching. These results suggest that GH has two important actions in this respect: during OGD, GH protects existing neurites, and during OGD/R stage, it promotes their growth, enhancing the endogenous neurorestorative mechanisms noticed in the OGD/R conditions. To explore the molecular mechanisms by which GH promoted neurite outgrowth when added during OGD injury, we also analyzed the expression of GAP-43 mRNA. As mentioned earlier, GAP-43 plays a role in axonal growth and branching [86]. Indeed, another report showed that the GAP-43 expression was upregulated in retinal ganglion cells after ischemia-induced damage [87]. As shown above, we observed that GAP-43 may play an important role in neurite regeneration, forming new neural circuits during OGD/R condition. Here, we presented, for the first time, that GH upregulated the GAP-43 mRNA expression in neurons exposed to OGD conditions, suggesting that GH facilitated neurite outgrowth in cultured hippocampal injured neurons via this pathway.

The administration of GH induced a significant increase in the mRNA expression of NRXNs and NLG1, implying beneficial effects upon synaptic plasticity in the OGD injured neurons. Interestingly, a stronger upregulation of these synaptic markers was observed when GH was added during OGD/R. Considering that NRXNs and NLG1 are important for the generation of long-term potentiation (LTP), which is positively regulated by GH [88] and is associated with synaptic plasticity and cognitive processes, our results may contribute to explain the improvements in cognitive functions that have been reported in animal models [27, 28, 85], GH-deficient patients [89], and brain-injured patients who receive GH replacement therapy [6, 13–15].

Initially, we hypothesized that the partial recovery observed in several parameters when the OGD injured hippocampal neurons were exposed to reoxygenation and normal glucose conditions (OGD/R) could be explained by the activation of an endogenous mechanism of neuroprotection and neuroplasticity mediated by neurotrophic factors such as BDNF, NT-3, IGF-1, and BMP4, whose expression was upregulated in response to hypoxia-ischemia injury. Remarkably, the addition of GH to the harmed cultures upgraded this endogenous recovery response to levels similar to those found under normoxic conditions. Thus, it is possible that GH acts together with the local neurotrophic factors to repair the damage caused by OGD conditions. To analyze this further, we evaluated the effect of GH treatment on BDNF, NT-3, IGF-1, and BMP4 mRNA expression under Nx, OGD, and OGD/R experimental conditions. We found that GH promoted a significant increase of these four neurotrophic factors under normoxic conditions, indicating that GH modulates them under physiological conditions. However, under OGD injury, only the BDNF expression was significantly upregulated by GH treatment. This is consistent with results where GH administration stimulated the synthesis of BDNF in animals with traumatic brain injury and improved cognitive functions [29]. Since BDNF promotes neuronal survival, neurite outgrowth, and synaptic function and plasticity [90], actions that are similar to those produced by GH in the present study, it is possible that the GH-stimulated BDNF expression during OGD conditions plays an important role in hippocampal neuron recovery. In contrast, this result was not mirrored under OGD/R conditions, since GH treatment provoked a marked reduction in the expression of BDNF and NT-3 mRNAs, suggesting their involvement in the recovery observed during the reoxygenation phase is under a complex regulation; these findings deserve further research. On the other hand, GH administration induced a significant upregulation of the BMP4 expression under both Nx and OGD/R conditions, but not when added during OGD injury. BMP4 has been associated with differentiation of neuronal stem cells (NSCs) and neuroprotection [91, 92]. Here, we provided evidence that this factor was induced by GH in the hippocampal neurons, an effect similar to that reported in the retinal tissue [12]. Considering that BMP4 promotes neurite outgrowth [93], it is possible that this neurotrophic factor acts together with GH to potentiate the restorative effect that was observed in the number and length of neurites in this study.

## 5. Conclusions

**Figure 10:**
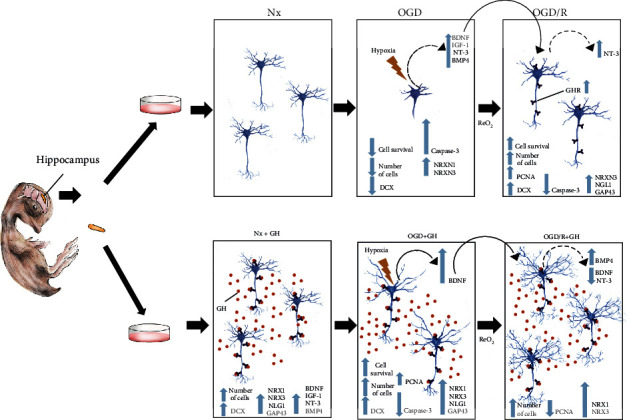
Infographic showing the effects of growth hormone (GH) on neuroprotection and neuroplasticity after OGD and reoxygenation (OGD/R) incubation conditions. Primary hippocampal neuron cultures obtained from chick embryos were maintained under normoxia conditions (Nx) for 5 days. Cell cultures were then exposed to oxygen-glucose deprivation (OGD) conditions for 24 h and then incubated under reoxygenation (OGD/R) conditions for another 24 h. Exposure to OGD injury significantly augmented apoptosis (caspase-3 activity) and reduced neurite outgrowth, cell survival, number of cells, and doublecortin immunoreactivity (DCX-IR), whereas the expression of synaptogenic markers (NRXN1, NRXN3) and neurotrophic factors (BDNF, NT-3, IGF-1 and BMP4) was increased. On the other hand, exposure of the harmed cultures to reoxygenation (OGD/R) triggered endogenous mechanisms that reversed most of the adverse effects of OGD and increased the expression of NT-3 and GHR. Treatment with growth hormone (GH) enhanced cell survival and neural plasticity in all incubation conditions, protecting the cultures from harm. Under Nx condition, GH addition (Nx + GH) increased the number of neurites, number of cells, DCX-IR, and expression of synaptogenic markers NRX1, NRX3, NLG1, and GAP43. Remarkably, treatment during OGD injury (OGD + GH) significantly reduced apoptosis, protected dendrites and neurites of hippocampal neurons, and significantly increased the level of several parameters, such as cell survival, the number of cells, DCX-IR, and PCNA expression, above those observed in the OGD/R condition. Also, GH stimulated the expression of BDNF and the synaptogenic markers NRXN1, NRXN3, NLG1, and GAP43. Furthermore, during the reoxygenation stage (OGD/R + GH), GH induced and enhanced plastic changes reflected in a significant increase in the number, length, and branching of the neurites, as well as in the expression of BMP4, NRX1, and NRX3, while the expression of PCNA, BDNF, and NT-3 was reduced.

As shown in Figure 10, this work provides evidence that, in response to OGD injury, reoxygenated hippocampal neurons are capable to activate endogenous mechanisms of neuroprotection and neuroplasticity that involve the local expression of several neurotrophins, and this in turn contributes to reverse some of the negative effects provoked by hypoxia-ischemia. This endogenous response is clearly enhanced by GH treatment, which protects from OGD injury and stimulates a significant increase in cell survival, exerts a clear antiapoptotic action, induces neurite outgrowth and branching, and promotes synaptogenesis, probably through a synergistic combination between its own effect and that of the local neurotrophic factors. This complex regulation deserves further investigation.

## Figures and Tables

**Table 1:** Antibodies.

Target	Host/type	Dilution	Source	Cat No.
DCX	Guinea pig/polyclonal	1 : 250 (ICC) 1 : 1000 (WB)	Millipore	AB2253
*β*-III-tubulin	Mouse/monoclonal	1 : 250	Abcam	Ab78078
GFAP	Rabbit/monoclonal	1 : 250	Cell Signaling	12389
*β*-Actin	Mouse/monoclonal	1 : 3000	Santa Cruz	SC-47778
Guinea pig IgG	Goat/Alexa fluor 488	1 : 1000	Invitrogen	A-11073
Mouse IgG	Goat/Alexa fluor 595	1 : 1000	Invitrogen	A-11032
Guinea pig IgG	Goat/HRP-conjugated	1 : 5000	Millipore	AP108P
Mouse IgG	Goat/HRP-conjugated	1 : 5000	Abcam	AB-20043

**Table 2:** Oligonucleotides.

Target	Primer	Sequence (5′-3′)	Size	Accesion number
cBDNF	Fwd Rev	AGCAGTCAAGTGCCTTTGGA TCCGCTGCTGTTACCCACTCG	167 bp	NM_001031616
cIGF1	Fwd Rev	TACCTTGGCCTGTGTTTGCT CCCTTGTGGTGTAAGCGTCT	170 bp	NM_001004384
cNT3	Fwd Rev	AGGCAGCAGAGACGCTACAAC AGCACAGTTACCTGGTGTCCT	248 bp	NM_001109762
cBMP4	Fwd Rev	CGCTGGGAGACCTTTGATGT CCCCTGAGGTAAAGATCGGC	153 bp	NM_205237.3
cGHR	Fwd Rev	ACTTCACCATGGACAATGCCTA GGGGTTTCTGCCATTGAAGCTC	180 bp	NM_001001293.1
cNRXN1	Fwd Rev	CCACTCTGATCATTGACCGGG CGCCAGACCTTCCACATAGT	392 bp	NM_001198975.1
cNRXN3	Fwd Rev	GCTGGGTCTCTCTTTGGGTC CACCCACAA AAAGGTCGCTG	394 bp	NM_001271923.1
cNLG1	Fwd Rev	CTCCAGTGTGTCCCCAGA AC CATCACAGGCTTAGGTCCCC	170 bp	NM_001081502.1
cGAP43	Fwd Rev	AGGAGCCTAAACAAGCCGAC TGCTGGGCACTTTCAGTAGG	178 bp	NM_001305054.1
C18S	Fwd Rev	CTCTTTCTCGATTCCGTGGGT TTAGCATGCCAGAGTCTCGT	100 bp	M59389

## Data Availability

The data used to support the findings of this study are included within the article. If further information is needed, it can be available from the corresponding author upon request.

## Supplementary Materials

Supplementary Materials Immunofluorescence characterization of primary hippocampal cell cultures showing the relative proportion of neurons (DCX-IR) and glial cells (GFAP-IR) at different culture times.
