# Supplementary material for: Growth Hormone (GH) Enhances Endogenous Mechanisms of Neuroprotection and Neuroplasticity after Oxygen and Glucose Deprivation Injury (OGD) and Reoxygenation (OGD/R) in Chicken Hippocampal Cell Cultures
Source: Neural Plast. 2021 Sep 16;2021:9990166. doi: 10.1155/2021/9990166 (PMC8461227; doi:10.1155/2021/9990166)
Supplement: Supplementary Materials — Immunofluorescence characterization of primary hippocampal cell cultures showing the relative proportion of neurons (DCX-IR) and glial cells (GFAP-IR) at different culture times. [file 9990166.f1.docx]

**Supplementary Figure 1**


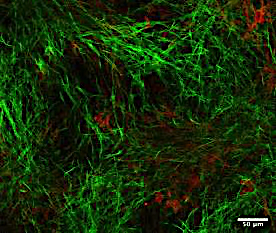

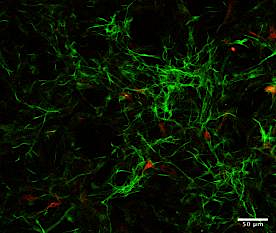


**DCX GFAP**


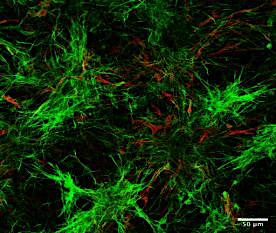


**6 d**

**D8**


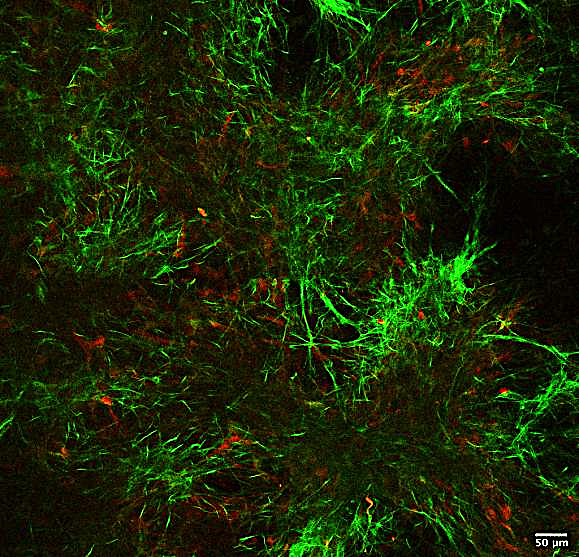


**8 d**

**2 d**

**4 d**

**i**

**ii**

**iii**

**iv**

**a)**

**b)**

**Figure S1. Characterization of primary hippocampal cell cultures.** (a) Representative immunofluorescence microphotographs of primary hippocampal cultures at different culture times: (i) 2 days, (ii) 4 days, (iii), 6 days, (iv) 8 days. Neurons were immunolabelled with fluorescent anti-DCX (green) antibody while glial cells with anti-GFAP (red) antibody, and the images were merged. Scale bar: 50 µm. (b) The relative proportions of DCX-IR and GFAP-IR fluorescence intensities were quantified by the number of pixels. Bars represent mean ± SEM. Asterisks (*) indicate significant differences between experimental groups (**, *p<* 0.01; **** *p<* 0.001) as assessed by two-way ANOVA and Tukey as *post-hoc* test. An unpaired Student’s t test was used to compare DCX-IR versus GFAP-IR fluorescence intensity at 2, 4, 6, and 8 days. The at sign (@) indicates significant differences. (@, *p* <0.05; @@, *p* <0.01).
